# Taking a closer look at mindful eating: incremental validity and importance of subfacets

**DOI:** 10.1007/s40519-022-01383-w

**Published:** 2022-03-17

**Authors:** Diana Peitz, Petra Warschburger

**Affiliations:** Counseling Psychology, University of Potsdam, Potsdam, Germany

**Keywords:** Mindful eating, Mindfulness, Maladaptive eating, Incremental validity, Subfacet

## Abstract

**Purpose:**

Mindful eating (ME) seems a promising approach to clarify the underlying mechanisms of mindfulness-based interventions for eating and weight-related issues. The current study aimed to investigate the incremental validity of this eating-specific approach beyond a generic conception of mindfulness and explore preliminary indication which subfacets of the multidimensional construct ME might be of particular importance in order to study them more precisely and tailor mindfulness-based interventions for eating and weight-related issues more properly.

**Methods:**

Self-report data (*N* = 292) were collected online. Hierarchical regression analyses were used to explore the incremental validity of ME beyond generic mindfulness, predicting maladaptive eating (emotional and uncontrolled eating) and consumption of energy-dense food. Multiple regressions were used to examine the impact of the seven different ME subfacets on the very same outcomes.

**Results:**

Findings demonstrated the incremental validity of ME on all outcomes. Generic mindfulness no longer predicted emotional eating, uncontrolled eating, or the consumption of energy-dense food when entering ME. The subfacet ‘*non-reactive stance*’ predicted all three outcomes significantly. For emotional and uncontrolled eating, the subfacets ‘*accepting and non-attached attitude toward one’s own eating experience*’, ‘*eating in response to awareness of fullness*’, *and the* ‘*awareness of eating triggers and motives*’ additionally showed a significant influence*.*

**Conclusion:**

ME seems a valuable approach in clarifying how mindfulness might impact eating and weight-related issues. Beyond that, it might be beneficial for upcoming interventions to strengthen specific ME subfacets, depending on the focused outcomes.

**Level of evidence:**

Level V, descriptive cross-sectional study.

## Introduction

Maladaptive or non-homeostatic eating (i.e., eating for reasons other than hunger) has shown to be associated with the development and maintenance of obesity and eating disorders (ED) [1–5]. Mindfulness-based interventions have demonstrated to reduce maladaptive eating behaviors (such as emotional eating or uncontrolled overeating) in persons across different weight groups [6, 7] and among subclinical and clinical ED samples [8, 9]. Thus, mindfulness became popular in research and practice to enrich the treatment of obesity and ED [10, 11].

Despite its popularity, the underlying mechanisms of mindfulness (resp. paying attention to the present moment without judging; [12]) in the context of eating seem so far largely unknown [6, 11, 13]. Some approaches focus on the attentive component of mindfulness by reducing the amount of food eaten while being with all senses with the food [14] or suppose that mindfulness might disrupt habit loops of maladaptive eating (or what the authors called “reward-related eating”) and facilitate rewiring eating-related learning processes [15]. Moreover, further preliminary attempts assume that mindfulness may operate through increased awareness of physical hunger and satiety cues as well as increased awareness of and reduced responsiveness to external and emotional cues [7]. Other assumed mechanisms refer to the mindfulness-immanent quality to be aware of the experience in the present moment (*present moment awareness*), the non-judgment or *acceptance* of this experience, and a *non-reactive stance toward this experience*, so called decentering [13]. However, none of these mechanisms have been sufficiently investigated to date. Regarding the increasing number of intervention studies, exploring the underlying mechanisms is crucial to tailor treatments properly in future research and practice [11].

Hitherto, it is unknown if mindfulness skills can be generalized to different domains of life [16]. There is even evidence that non-eating-specific mindfulness interventions did not affect maladaptive eating [17]. Thus, applying an eating-specific approach seems beneficial to utilize mindfulness in the context of eating behavior and study underlying mechanism in this evolving field [16, 18, 19]. This is one reason why recent research has focused on the context-specific *Mindful Eating* (ME) approach.

In line with generic mindfulness [20], ME is conceptualized as a multidimensional construct [7, 21]. Using comprehensive factor analyses, Peitz and colleagues. [18] identified seven subfacets and consecutively operationalized ME as “bringing an accepting and non-attached attitude to the experience of eating (1) while deliberately paying attention (2) to the present moment with all senses (3), being aware of not only motives and needs which trigger eating (4) without directly reacting to them (5) but also integrating this knowledge with the awareness of physiological hunger and satiety signals to guide one’s own eating behavior consciously (6). Additionally, ME includes the awareness of connectedness between the earth and all living beings setting the process of eating in a broader picture (7)” ( [18], p. 12).

Several correlational studies have shown significant negative associations between higher ME and both maladaptive eating behaviors and less healthy nutrition behavior (e.g., [16, 22–25]). However, only one study investigated the assumed incremental validity of ME over generic mindfulness: Beshara and colleagues [26] (using hierarchical regression analyses on cross-sectional data) showed ME to fully mediate the relationship between the generic construct and the self-reported serving size of energy-dense food, which is associated with weight gain and binge eating episodes.

Moreover, there is preliminary evidence that certain ME subfacets are more strongly associated with particular eating behaviors than others [27–29]. Thus, they might be of particular interest in preventing and treating eating and weight-related issues. To approach the mechanism of action, it is crucial to identify those subfacets of a multidimensional construct that in fact have predictive power (and distinguish them from those with non-predictive power); otherwise, the effect of the overall construct on the outcome to be addressed may be under- or overestimated [30]. Previous studies used different instruments to assess ME, sharing some subfacets but differing in others. To compare all subfacets, empirically gauging their specific importance, the use of a comprehensive measure assessing all subfacets of ME seems pivotal.

**Aims of the current study:**

1. ME seems a promising approach to study the underlying effects of mindfulness on eating behaviors that are associated with the development and maintenance of obesity and ED. However, the incremental validity of ME over generic mindfulness in this context has not been sufficiently investigated. Thus, the current study aims to explore if ME explains variance in eating behaviors associated with obesity and ED, i.e., maladaptive eating (emotional eating, uncontrolled eating) and, consumption of energy-dense food, beyond the generic construct.
2. ME has shown to be a multidimensional construct. In approaching the mechanism of action, it seems essential to explore whether and which subfacets of ME have the most impact on the previously mentioned variables and thus seem of particular interest in treating eating and weight-related issues.

## Methods

### Procedure and participants

Data were gathered online within the validation of the final version of the Mindful Eating Inventory (MEI) [18]. The convenience sample was recruited mainly via social media and specific websites and mail distributors regarding eating behavior. Participation was voluntary, and participants received an incentive (ME information booklet; opportunity to take part in a lottery). After providing obligatory demographic information (e.g., date of birth, height, and weight), individuals answered questions on eating behavior and socioeconomic status.

Individuals were included in the study if they were at least 18 years of age, provided informed consent, and completed the survey. The full sample consisted of 292 individuals (81% female) with a mean *Body Mass Index* (BMI = weight(kg)/height(m)^2^; 31) of *M* = 25.7 (*SD* = 6.7, range 18.5–59.4). Age ranged from 18.3 to 65.8 years (*M* = 34.7, *SD* = 11.4). According to Winkler Index [32], 18% could be assigned to a lower socioeconomic class, 63% to a middle and 19% to the upper class.

### Measures

*ME* was assessed with the *Mindful Eating Inventory* (MEI) [18]. The MEI is a comprehensive instrument that unites the different ME subfacets detected in previously published scales within one inventory. It assesses the multidimensional construct of ME with seven subfacets (internal consistency in brackets): (1) ‘*A*ccepting and *N*on-attached *A*ttitude toward one’s own eating experience’ (ANA; *α* = 0.87) with five items (e.g., “I accept my eating behavior as it is right now.”), (2) ‘*A*wareness of *S*enses while *E*ating’ (ASE; *α* = 0.86) with five items (e.g., “I taste every bite of food that I eat”), (3) *E*ating in *R*esponse to awareness of *F*ullness (ERF; *α* = 0.86) with five items (e.g., “I stop eating when I’m full, even if my plate is not empty yet.”), (4) ‘*A*wareness of Eating *T*riggers and *M*otives’(ATM; *α* = 0.83) with four items (e.g., “I’m able to notice if I’m physically hungry or if I want to eat for other reasons (e.g., boredom, habit, availability, etc.).”), (5) ‘Inter*con*nectedness’ (CON; *α* = 0.82) with three items (e.g., “When I’m eating, I bring to mind where my food comes from and how it came to me.”), (6) ‘*N*on-*R*eactive *S*tance’ (NRS; *α* = 0.76) with our items (e.g., “While I eat, I focus all my attention on the food.”, reversed), and (7) *F*ocused *A*ttention on *E*ating’ (FAE; *α* = 0.81) with four items (e.g., “While I eat, I focus all my attention on the food.”). The 30 items were rated on a six-point scale ranging from *almost never* to *almost always.* Internal consistency of the total score was *α* = 0.92 in the current sample.

The *Comprehensive Inventory of Mindfulness Experiences* (CHIME) [33] was applied to assess *generic mindfulness*. The 37 items (8 subscales) of the CHIME are assessed on a six-point scale ranging from *almost never* to *almost always*. For this study, the total score was used (internal consistency in current sample: *α* = 0.92).

*Emotional eating* and *uncontrolled eating* were measured by an 18-item version of the *Three Factor of Eating Questionnaire* (TFEQ-R18V2) [34]. Items were taken from the *Fragebogen zum Essverhalten (FEV)* [35], the German equivalent of the TFEQ. The TFEQ-R18V2 measures three domains of eating behaviors—together with cognitive restraint—on a four-point scale with alternating scale point descriptions. In the current sample, Cronbach’s alpha reached *α* = 0.94 for emotional eating and *α* = 0.90 for uncontrolled eating.

In accordance with Beshara and colleagues [26], we assessed the *frequency of consuming energy-dense food.* Participants were asked how often they consumed (1) ‘fast food (such as burger, pommes, kebab, hot dogs) or instant meals (such as lasagna, pizza)’ and (2) ‘sweets or snacks (e.g., chocolate, potato chips, ice cream, cake, pudding)’. Answers ranged on a six-point rating scale (‘never/seldom’, ‘1–3 times a month’, ‘1–2 times a week’, ‘several times a week’, ‘daily’, ‘several times a day’).

### Analyses

First, Pearson’s product moment correlations were computed between ME, generic mindfulness, and the three outcomes emotional eating, uncontrolled eating, and consumption of energy-dense food to prove their associations for further analyses.

Second, hierarchical multiple regressions were computed. Generic mindfulness was entered first (first model), while ME was entered second (second model) to investigate incremental validity of ME above and beyond generic mindfulness.

Third, multiple regression analyses were used to investigate possible impact of the single ME subfacets (entered simultaneously) over and above each other regarding emotional eating, uncontrolled eating, and consumption of energy-dense food and to identify predictive facets for these outcomes.

All analyses were performed with SPSS 26. There were no missing data regarding the investigated variables.

## Results

ME and generic mindfulness were correlated significantly positively (*r* = 0.61; *p* < 0.001). Correlations showed significant negative associations between the ME total score, emotional eating, uncontrolled eating, and consumption of energy-dense food. Generic mindfulness was significantly negatively associated with emotional eating, uncontrolled eating, and consumption of energy-dense food, but to a lesser degree. Detailed correlation values can be found in Table 1.

**Table 1:** Correlation between Generic Mindfulness, Mindful Eating and Maladaptive Eating and Nutrition Behaviors

	Emotional Eating	Uncontrolled Eating	Energy-dense food consumption
CHIME	− 0.45*	− 0.45*	− 0.21*
ME_total_	− 0.67*	− 0.75*	− 0.36*
ANA	− 0.58*	− 0.50*	− 0.18
ASE	− 0.33*	− 0.36*	− 0.27*
ERF	− 0.50*	− 0.68*	− 0.27*
ATM	− 0.44*	− 0.48*	− 0.20*
CON	− 0.18	− 0.24*	− 0.17
NRS	− 0.59*	− 0.70*	− 0.34*
FAE	− 0.38*	− 0.41*	− 0.24*

*Note. CHIME* = Comprehensive inventory of mindfulnes experience, *MEI*_*total*_ = Mindful Eating total score, *ANA* = Accepting and Non-attached Attitude toward one’s own eating experience, *ASE* = Awareness of Senses while Eating, *ERF* = Eating in Response to awareness of Fullness, *ATM* = Awareness of eating Triggers and Motives, *CON* = Interconnectedness, *NRS* = Non-Reactive Stance, *FAE* = Focused Attention on eating. *p < 0.001

Hierarchical regression analyses showed that generic mindfulness significantly predicted emotional eating (*β* − 0.447; *p* < 0.001). This first model explained 20% of the variance. The inclusion of ME (*β* − 0.631; *p* < 0.001) enhanced the explained variance to 45%, indicating incremental validity. When entering ME, generic mindfulness no longer predicted emotional eating (*β* − 0.066; *p* = 0.234).

Regarding uncontrolled eating, generic mindfulness explained 20% of the first model and significantly predicted the construct (*β* − 0.449; *p* < 0.001). When entering ME (*β* − 0.751; *p* < 0.001) in the second model, the explained variance increased to 56%. Generic mindfulness did no longer predict uncontrolled eating (*β* − 0.006; *p* = 0.911) in this model.

Generic mindfulness significantly predicted the consumption of energy-dense food (*β* − 0.211; *p* < 0.001) and explained 4% of its variance. When entering ME (*β* − 0.369; *p* < 0.001), the explained variance increased to 12%. Consumption of energy-dense food was no longer predicted by generic mindfulness in this second model (*β* 0.013; *p* = 0.855).

Multiple regression analyses revealed that entering all seven ME subscales together explained 53% of the variance in emotional eating, 69% of the variance in uncontrolled eating, and 14% of the variance in the consumption of energy-dense food. ANA, ERF, ATM and NRS significantly predicted both emotional eating and uncontrolled eating, NRS significantly predicted the consumption of energy-dense food (see Table 2).

**Table 2:** Multiple Regression Analyses of ME Subfacets

	Criterion: Emotional Eating		Criterion: Uncontrolled Eating		Criterion: Energy-dense food consumption	
	*β*	*p*	*β*	*p*	*β*	*p*
ANA	− 0.323	< 0.001	− 0.151	< 0.001	.009	0.890
ASE	− 0.008	0.889	0.016	0.722	− 0.147	0.055
ERF	− 0.150	0.003	− 0.369	< 0.001	− 0.073	0.284
ATM	− 0.132	0.008	− 0.110	0.006	.009	0.895
CON	− 0.008	0.869	− 0.046	0.235	− 0.024	0.702
NRS	− 0.315	< 0.001	− 0.415	< 0.001	− 0.260	< 0.001
FAE	− 0.095	0.057	− 0.057	0.155	− 0.057	0.397

*Note: ANA* accepting and non-attached attitude toward one’s own eating experience, *ASE* awareness of senses while eating, *ERF* eating in response to awareness of fullness, *ATM* awareness of eating triggers and motives, *CON* interconnectedness, *NRS* non-reactive stance, *FAE* focused attention on eating, *β* = standardized *β* weights,* p* = *p* values (corrected for multiple testing)

## Discussion

In our study, a multidimensional eating-specific mindfulness approach (ME), showed incremental value beyond generic mindfulness regarding maladaptive eating behaviors and the consumption of energy-dense food. Furthermore, the single ME subfacets contribute to a different extent to these outcomes. This observation might be of particular interest in tailoring mindfulness-based interventions for eating- and weight-related issues.

### Maladaptive eating behaviors

Both generic mindfulness and ME were significantly negatively correlated with maladaptive eating behaviors. However, entering ME as a predictor not only improved the explained variance, but also the significant prediction of generic mindfulness on both emotional and uncontrolled eating faded. ME remained the only significant predictor. Our results indicate the incremental validity of ME above and beyond generic mindfulness in the context of maladaptive eating behaviors. Furthermore, they give preliminary evidence that ME might work as a mediator between those constructs. Consequently, future research with longitudinal data must show if ME (and its subfacets) is one missing piece in clarifying the mechanism of action regarding mindfulness-based interventions in the context of eating and weight-related issues.

Within a second step, all seven subfacets were simultaneously entered in a multiple linear regression model. They explained more than 50% of the variance in emotional eating and almost 70% of the variance in uncontrolled eating. As both of these eating patterns are assumed to foster the development and maintenance of ED [1, 5], results give initial evidence that targeting them with ME-based interventions might be beneficial. In this context, the following four ME subscales explained the significant amount of variance in both maladaptive eating behaviors and should therefore be focused on while targeting these behaviors in pre- and intervention: *accepting and non-attached attitude toward one’s own eating experience *(*ANA*)*, eating in response to awareness of fullness *(*ERF*)*, the awareness of eating triggers and motives *(*ATM*)*,* and a *non-reactive stance *(*NRS, i.e., meaning an observing, non-impulsive attitude toward eating triggers*).

In explaining uncontrolled eating, particularly NRS and ERF seemed important, as they depict counterparts of this behavior. Instead of losing control over one’s own eating behavior, which often leads to overeating [5], NRS and ERF describe self-regulated eating skills in line with physiological needs. Emphasizing particularly these ME skills in interventions might contribute to the enhancement of a conscious, self-determinant regulation of eating behavior. As potential mediators, NRS and ERF might explain the positive influence of generic mindfulness on maladaptive eating behaviors found in intervention studies [7, 10]. Regarding emotional eating, again, NRS explained a high amount of variance. This result seems reasonable, as NRS describes a conscious way of handling triggers like emotional cues instead of simply reacting to them (e.g., with eating; [18]).

Also, ATM explains a significant amount of variance in emotional as well as uncontrolled eating. This finding indicates that being aware of triggers might facilitate eating according to physiological needs (ERF, third important subfacet) and it is in line with assumptions on reducing reward-related eating through mindfulness by the working group of Brewer and colleagues [15]: The authors argue that the awareness of eating triggers is the first step in changing habitual maladaptive eating patterns. The particular importance of ATM in predicting uncontrolled and emotional eating behaviors in our study could possibly also explain the paradoxical findings regarding the moderating effects of ME on the relationship between emotional functioning and eating styles in overweight and obese women in a recently published study [36], as this subfacet was not sufficiently captured by the ME instrument used by the working group.

However, our results showed that particularly ANA seems of special impact in explaining maladaptive eating. These findings might explain the assumption that a non-judgmental stance toward one’s own eating experience may interrupt dysfunctional eating circles associated with overeating [37]. More specifically, by accepting the emotional or uncontrolled eating behavior, ANA may buffer the effects of high self-criticism endangering dysfunctional circles that lead to more overeating [38, 39].

Regarding the importance of certain ME subfacets in the context of prevention and treatment of eating and weight-related issues, our findings are in line with the aforementioned theoretical assumptions [7, 13] and results: the ME acceptance subdomain (equivalent to ANA) and the ME non-reactivity subdomain (equivalent to NRS) were found to be particularly strongly correlated with maladaptive eating behaviors [23, 29]. Also, *intuitive eating* (i.e., eating in line with physiological needs), an adaptive eating style that can be considered as equivalent to ERF within the ME framework [18], was associated with healthy eating habits comparable to ME [40].

### Consumption of energy-dense food

In line with the results of Beshara and colleagues [26], who first showed incremental validity of ME beyond generic mindfulness in the context of eating and according to whom we chose to assess a food consumption outcome to facilitate comparability, ME not solely showed an increase in the explained variance of the consumption of energy-dense food; this outcome was no longer predicted by generic mindfulness when ME was added. Our findings align well with former results on higher ME and healthier food choices from correlational studies [22, 27, 41] and experiments [42, 43], and indicate that ME might support that “unhealthy foods” become less attractive [44].

In comparison to maladaptive eating behaviors, ME explained less variance in consumption of energy-dense food. Future prospective studies should investigate more deeply if our results indicate that eating-specific mindfulness may have a greater impact on *how* rather than *what* to eat. In our study, only one subfacet (NRS) proved to be a significant predictor of the consumption of energy-dense food. Hutchinson and colleagues [27] found the awareness component of ME to be the most important subfacet in predicting maternal dietary choices. A meta-analysis [14] showed comparable effects of eating attentively with all senses on the amount of food eaten. In our study, the equivalent ASE (being there with all senses whilst eating) was next to NRS the second important predictor, but yielded no significance. This might be due to our only focus on so-called “unhealthy foods”. Our findings should be further investigated in upcoming studies using different assessments.

## Strengths and limitations

Our sample contained a wide range of age, socioeconomic status and especially BMI, representative of the German population [45]. However, the sample comprised mainly females. Further studies should stress the inclusion of more males to generate more generalizable results in terms of this issue.

Our study enables the comparability with the only other result of ME’s incremental validity provided by Beshara and colleagues [26]. Though, particularly with respect to this result, one should consider that we only used two questions on the consumption of energy-dense food, reflecting only a narrow but also an essential part of our food intake. Future studies should use more elaborate food frequency questionnaires to investigate the influence of mindfulness on nutrition behavior more deeply.

We used a comprehensive ME measure that was able to compare the impact of different subfacets. Based on our cross-sectional design, causal conclusions can only be hypothesized. Prospective and experimental studies are warranted to broaden our knowledge on ME, especially regarding the predictive power and potential mediating effects of the different ME subfacets and their significance in treating obesity and ED.

## Preliminary clinical suggestions

In line with other findings [17], our results give a preliminary implication that maladaptive eating and nutrition behaviors might be addressed more beneficially by mindfulness exercises or interventions with a specific focus on eating, such as MB-EAT [46] or *Mindful Eating - Conscious Living* (ME-CL) [47]. Thus, these context-specific interventions should be more focused on clinical practice and more extensively studied in research than to date [13, 48].

Moreover, findings on the subscale level indicate that exercises regarding the enhancement of a non-reactive stance toward eating (NRS; resp. solely being aware of eating triggers without reacting to them), might be beneficial. So-called “mini meditations” [46] before starting to eat may decrease maladaptive eating behavior as well as “unhealthy” nutrition behavior, since NRS showed significant predictions of both behaviors.

For maladaptive eating, such as emotional eating and/or uncontrolled eating, there seems to be more mechanisms at play, and, thus, they should be addressed in interventions via different exercises. For instance, the ME-CL core exercise ‘*9 HUNGER*’ [47] aims to enhance the ability to become aware of and distinguish between different eating triggers and motives (ATM subfacet) in order to decide if the body needs food or one wants to eat, e.g., for emotional reasons. This aims to facilitate a conscious decision on food intake and caring of emotional needs in other ways than food. Especially for clinical groups with elevated emotional and/or uncontrolled eating, this exercise might be particularly helpful. However, these and other assumptions need to be researched in future intervention studies and experiments.

## Conclusion

In sum, ME seems a valuable approach to approximate the underlying mechanisms of mindfulness-based interventions on obesity and ED, as it possesses incremental validity beyond the generic construct on maladaptive eating and the consumption of energy-dense food, both of which have been shown to be associated with the development and maintenance of these clinical pictures. Furthermore, our findings indicate that different subfacets might be important for the treatment of eating and weight-related issues, depending on the primary goal of the intervention. Further longitudinal studies with clinical samples are needed to find out more about the potential of ME and its subfacets in explaining the underlying mechanisms of mindfulness-based interventions to target them more precisely.

**What is already known on this subject?**

- Mindful Eating (ME) is suggested as one mechanism to explain the effects of mindfulness-based interventions on eating and weight-related issues.
- Hitherto, evidence for its incremental validity on maladaptive eating and nutrition behaviors beyond generic mindfulness and the impact of certain ME subfacets on these behaviors is mainly missing.

**What this study adds?**

- This study provides initial evidence that ME might be helpful to study the underlying mechanisms of mindfulness in the context of eating. It explained significantly more variance in maladaptive eating behaviors and the consumption of energy-dense food than the generic construct.
- Depending on the outcomes, particular ME facets seem of interest for tailoring mindfulness-based interventions for obesity and eating disorders properly.

## Data Availability

The datasets generated during and/or analyzed during the current study are available from the corresponding author on reasonable request.
